# Supplementary material for: A TetR-family transcription factor regulates fatty acid metabolism in the archaeal model organism Sulfolobus acidocaldarius
Source: Nat Commun. 2019 Apr 4;10:1542. doi: 10.1038/s41467-019-09479-1 (PMC6449355; doi:10.1038/s41467-019-09479-1)
Supplement: Supplementary file 1 — Supplementary Information [file 41467_2019_9479_MOESM1_ESM.pdf]

SUPPLEMENTARY INFORMATION

A TetR-family transcription factor regulates fatty acid metabolism in the archaeal model organism *Sulfolobus acidocaldarius*

Wang et al

Supplementary Note 1. Detailed description of $FadR_{Sa}$-DNA contacts in the cocrystal structure.

A map of the observed protein-DNA interactions illustrates the extensive number of contacts that are established between each of the two DNA duplexes and the three interacting $FadR_{Sa}$ monomers (Figure 4a; Supplementary Data 1). These contacts take place in the major groove of the DNA and involve mainly $FadR_{Sa}$ residues from the α3 recognition helix (Tyr51, Phe52 and Tyr53) and from the loop between α2 and α3 (Ala46, Tyr47, Gly48 and Leu49). They consist of backbone contacts with the sugar or phosphate moiety in addition to base-specific contacts with 13 of the 21 bps of each DNA duplex. Tyr53 and Lys57, highly conserved in FadR-like or TetR-like proteins, respectively (Supplementary Figure 5a), form hydrogen bonds with the phosphate backbone thereby anchoring $FadR_{Sa}$ to the DNA so that the recognition helices are appropriately oriented for interactions. The base-specific contacts made by each of the $FadR_{Sa}$ monomers are essentially established by identical amino acid residues although the local nucleic acid environment encountered by each of the monomers is diverse (Figure 4b; Supplementary Data 1). The majority of the base-specific contacts are hydrophobic with involvement of Tyr47, Leu49, Tyr51 and Phe52: they form a hydrophobic plane docking into the major groove (Figure 4c). It can be postulated that the contacts established between Leu49, Tyr51 and Phe52 on one hand and methyl groups of thymines on the other hand (T5, T6, T11, T12, T13 and T17 of DNA chain X; T2, T7 and T14 of DNA chain Y; Figure 4b) endow a certain degree of sequence specificity to the interaction. Besides the hydrophobically interacting residues, Gly48 of subunit B forms a hydrogen bond between its amide nitrogen and the N7 of a guanine (Figure 4b; Supplementary Data 1). Although not categorized as hydrogen bonds, weak electrostatic interactions also exist between the N7 atom of G7 of the X chain and Gly48 of subunit A thereby categorizing it as a sequence-specific contact for all subunits. Besides protein-DNA contacts, a protein-protein interaction was also observed in the cocrystal structure between $FadR_{Sa}$ dimers bound on different sides of the DNA helix involving Asn37. The orientation of this residue in opposing monomers (e.g. subunits E and B) enables the formation of weak electrostatic interactions that could further stabilize the protein-DNA complex. The establishment of this protein-protein interaction might be possible because of the relative orientation of the two DNA-bound dimers to each other. This relative orientation is characterized by an angle of 122°, which is smaller than other TetR proteins that bind as a pair of dimers (Supplementary Figure 5b).

Supplementary Note 2. Detailed description of the interpretation of RNA-seq and qRT-PCR analyses.

Quantitative reverse transcriptase PCR (qRT-PCR) confirmed higher expression levels in the Δ*$fadR_{Sa}$* strain for a subset of the *Saci_1103-Saci_1126* genes, including the *Saci_1105*, *Saci_1123* and *Saci_1124* genes, that were not reaching the significance threshold in the RNA-seq analysis (Figure 6b). The lack of detecting regulation of the CopG regulator-encoding *Saci_1124* with RNA-seq is explained by the gene being expressed at very low, almost undetectable levels. Nevertheless, qRT-PCR analysis demonstrated that the expression of this gene is 2.74 fold higher in the *$fadR_{Sa}$* mutant strain (with a *P*-value of 0.0013) (Figure 6b). We can therefore conclude that $FadR_{Sa}$ is a local regulator of the entire *Saci_1103-Saci_1126* gene cluster (Figure 6a). RNA-seq analysis did not reveal differential expression for any of the genes adjacent to the other ChIP-seq identified genomic binding regions. However, several other genes were found to have a slightly lower expression in the Δ*$fadR_{Sa}$* strain, pointing to an indirect regulatory effect (Supplementary Data 2). These genes include an operon encoding a putative sulfate reduction pathway (*Saci_2198-Saci_2203*) and cytochromes (*Saci_1858*, *Saci_1859* and *Saci_1861*). The observation that several of these genes are transcribed in operons strengthens the assumption that the observed small expression changes are relevant despite the determined fold-changes being unreliable (as confirmed by qRT-PCR (Figure 6b)).

Supplementary Note 3. Description of conformational differences in $FadR_{Sa}$ dimers in the $FadR_{Sa}$-DNA cocrystal structure.

Intriguingly, the central (subunits E and F) but not the two flanking dimers (subunits A, B, C and D) in the asymmetric unit of the $FadR_{Sa}$-DNA structure harbored additional electron density in the ligand-binding pockets (Figure 3a). The exact nature of these ligands is unknown, although they are modeled to contain a CoA moiety. This can be explained by conformational differences in the central *versus* flanking dimers, which is also reflected in the distance between the two α3 recognition helices, which is 36.8 Å and 37.1 Å for the flanking dimers AB and CD, respectively, and much larger, 45.3 Å, for the central dimer (EF). The latter resembles the conformation of lauroyl-CoA-bound $FadR_{Sa}$, in which recognition helices are separated by 43.2 Å. This indicates that the ligand-bound E and F subunits of the central dimer are not well-positioned for simultaneous interaction with major groove segments of a single DNA molecule but that DNA binding can be accommodated for the two α3 helices by two distinct DNA duplexes. This creates an artificial situation with $FadR_{Sa}$ in a single complex both having ligand molecules bound and being bound to DNA at the same time.

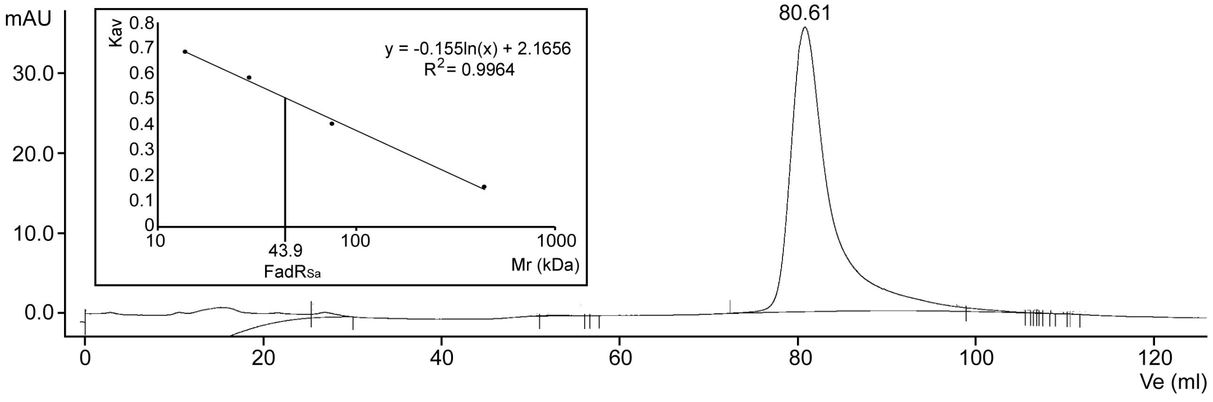

Supplementary Figure 1. Oligomeric state of FadR$_{Sa}$ in solution. The oligomeric state of native FadR$_{Sa}$ was analyzed by size exclusion chromatography using a Hiload 16/600 Superdex 200 pg column (GE Healthcare) equilibrated with 0.05 M sodium phosphate buffer (pH 7.4) containing 0.15 M NaCl. The calibration curve shown in the inset was prepared with ribonuclease A (13.7 kDa), carbonic anhydrase (29 kDa), conalbumin (75 kDa) and ferritin (440 kDa). After injecting 0.5 ml of purified recombinant FadR$_{Sa}$ at a concentration of 2.5 μg ml^{-1}, a single peak was observed with an elution volume (Ve) of 80.61 ml, corresponding to a molecular weight of 43.9 kDa as indicated on the calibration curve. Considering a molecular weight of 24 kDa for a His-tagged FadR$_{Sa}$ subunit, this observation supports a homogenous population of FadR$_{Sa}$ homodimers.

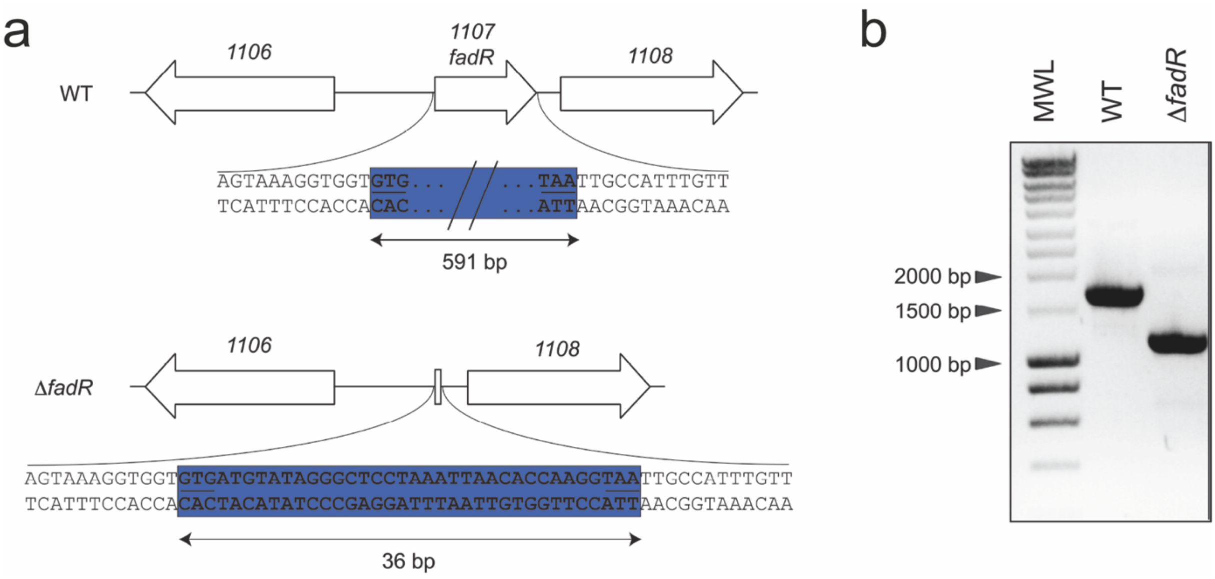

Supplementary Figure 2. Construction of an in-frame markerless FadR$_{Sa}$ deletion mutant. (**a**) Schematic representation of the genomic environment of the *fadR$_{Sa}$* gene in a *S. acidocaldarius* wild-type (MW001) and *S. acidocaldarius* MW001 Δ*fadR$_{Sa}$* strain, respectively. The length of the intact or remainder of the disrupted gene is mentioned, the latter resulting in a nonfunctional protein product of 12 amino acids. (**b**) PCR analysis of genomic DNA of the wild-type and Δ*fadR$_{Sa}$* strain with primers ep397 and ep398 (Supplementary Data 4) demonstrating a successful deletion. Product sizes are 1716 bp (WT) and 1161 bp (Δ*fadR$_{Sa}$*). MWL = molecular weight ladder.

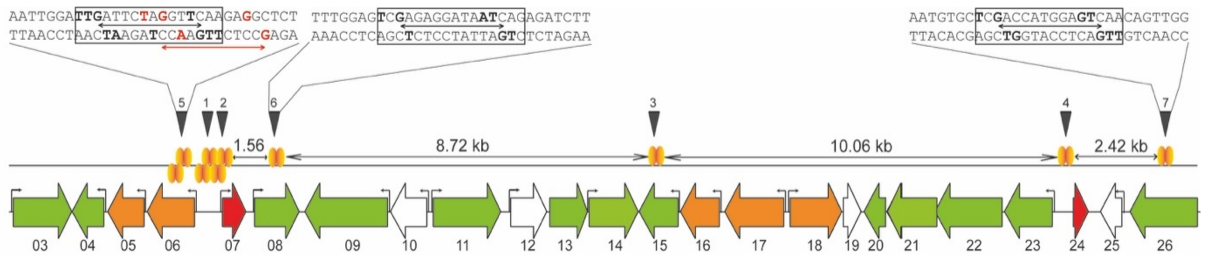

Supplementary Figure 3. Schematic overview of all (putative) FadR$_{Sa}$ binding sites in the *Saci_1103-Saci_1126* gene cluster. This scheme is depicted with indication of distances between them (in kb) and of whether the site is bound in a dimer or dimer-of-dimer mode (based on observations described in the section "DNA-binding stoichiometry of FadR$_{Sa}$"). Sequences of the newly predicted binding sites 5, 6 and 7 are shown, with indication of nucleotides that are assumed to be important for the interaction in bold (black = central operator; red = left or right operator for binding of second dimer).

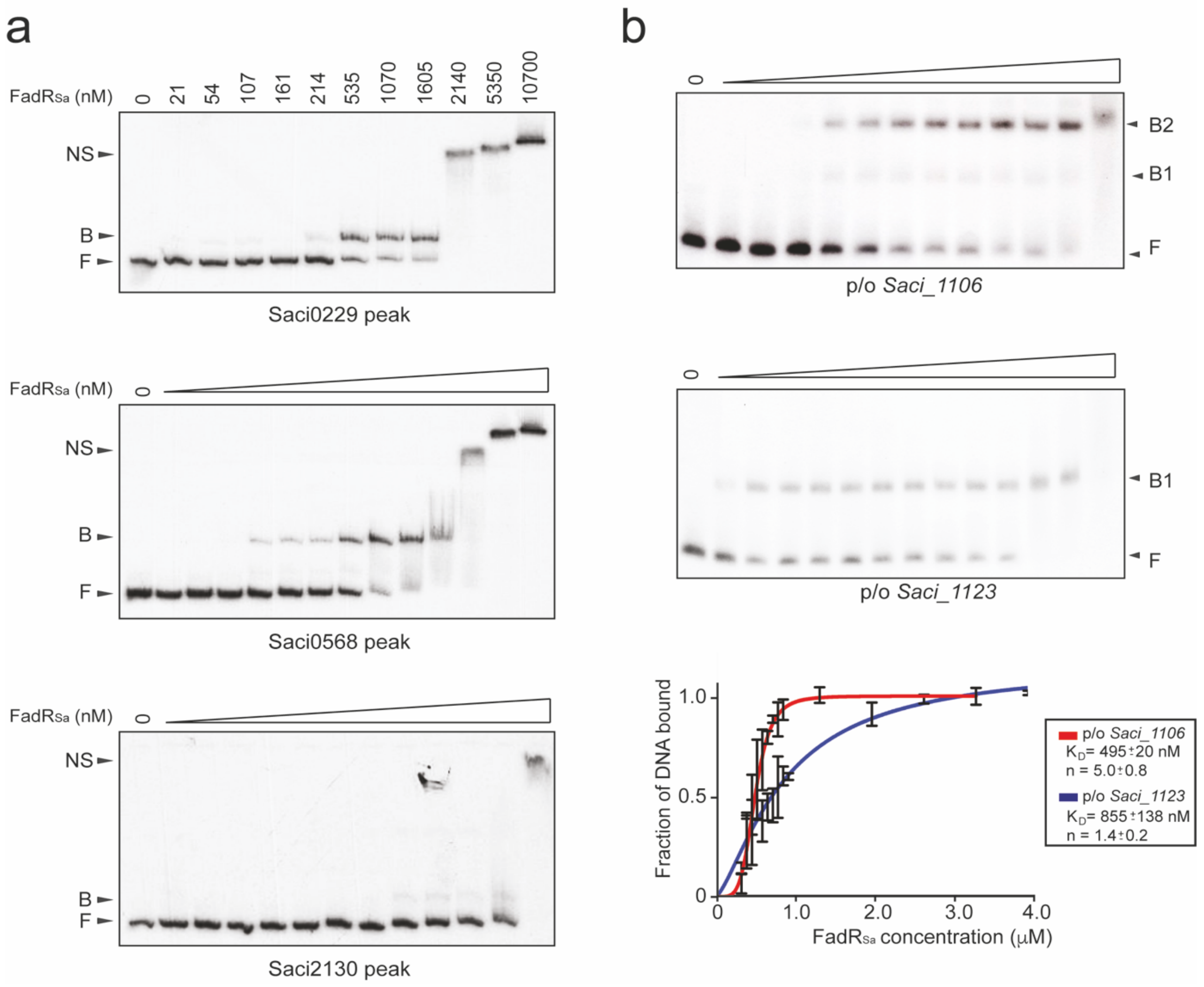

Supplementary Figure 4. *In vitro* DNA-binding analysis of FadR$_{Sa}$ to fragments representing ChIP-seq peaks. (**a**) Electrophoretic mobility shift assays (EMSAs) were performed with radiolabeled DNA probes representing high-enrichment binding regions identified in the ChIP-seq experiment located outside the lipid/fatty acid metabolism gene cluster (Supplementary Table 2). Experimental procedures are described in the Methods section. The same protein concentrations were used in the three experiments. Nucleic acid populations are indicated as follows: F = free DNA, B = specifically bound DNA, NS = non-specifically bound DNA. Note that for the Saci2130 peak fragment only a minor fraction of specific FadR$_{Sa}$-DNA complex was detected, which is hardly visible. (**b**) Binding parameter analysis of the interaction between FadR$_{Sa}$ and the promoter/operator (p/o) regions of *Saci_1106* and *Saci_1123*. Representative concentration-gradient EMSAs are shown that were used for the densitometric analysis and construction of binding curves. Below autoradiographs, binding curves are displayed that were fitted with a Hill equation on densitometric data with indication of the equilibrium dissociation constant K_D and Hill coefficient n as a measure for cooperativity. Averages are made for technical triplicates; error bars represent standard deviations.

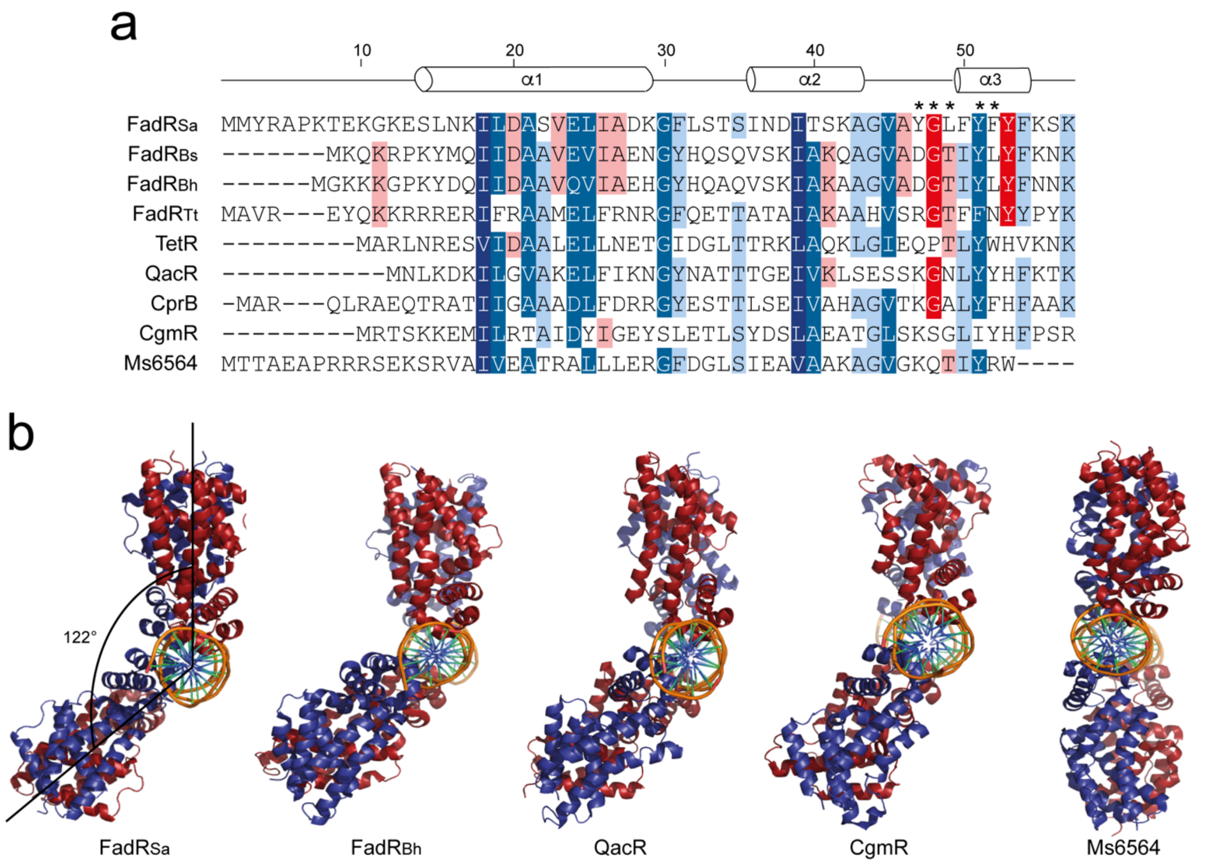

Supplementary Figure 5. Comparison of the DNA-binding mode of TetR family proteins. (**a**) Sequence alignment of the DNA-binding domains of the following TetR-like proteins: $FadR_{Sa}$ of *Sulfolobus acidocaldarius*, $FadR_{Bh}$ of *Bacillus halodurans*, $FadR_{Tt}$ of *Thermus thermophilus*, TetR of *Escherichia coli*, QacR of *Staphylococcus aureus*, CprB of *Streptomyces coelicolor*, CmgR of *Corynebacterium glutamicum* and Ms6564 of *Mycobacterium smegmatis*. Residues are indicated in blue when they are conserved in all (darkest blue), all but one (dark blue) or all but two (light blue) proteins (identical or similar residues). Residues are indicated in dark and light red when they are conserved in all or all but one FadR proteins, respectively (identical residues). Residue position numbers and secondary structure elements are indicated for $FadR_{Sa}$. Asterisk symbols denote $FadR_{Sa}$ residues in the DNA-binding domain that establish base-specific interactions. (**b**) Structural comparison of DNA-bound complexes of TetR-like proteins that bind in a dimer-of-dimer interaction mode (PDB codes: 6EN8 ($FadR_{Sa}$); 5GPC ($FadR_{Bh}$)[1]; 1JT0 (QacR)[2]; 2YVH (CgmR)[3]; 4JL3 (Ms6564)[4]). Each monomeric subunit of a dimer is colored differently (red or blue). In the case of $FadR_{Sa}$, only a single DNA molecule (XY) is shown with its two interacting dimers AB and EF.

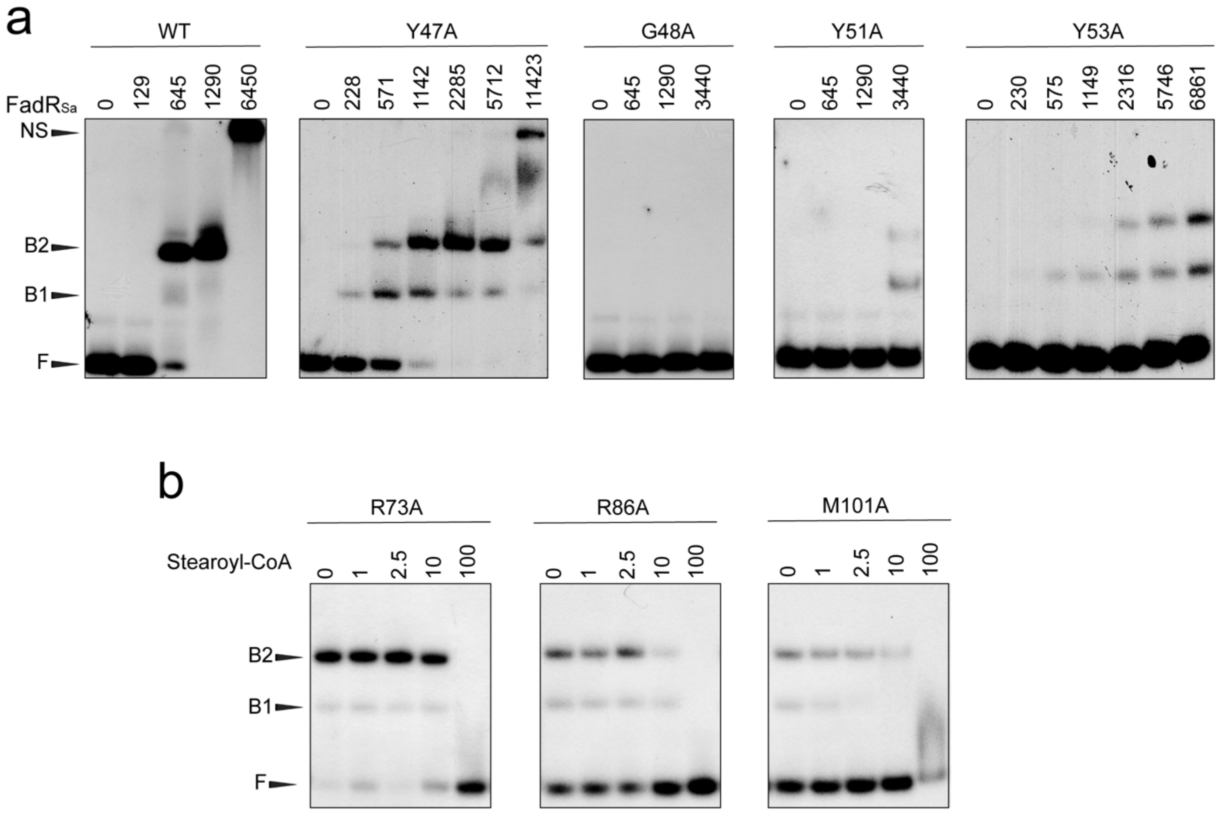

Supplementary Figure 6. ***In vitro*** **DNA-binding and ligand-response analysis of FadR$_{Sa}$ mutants.** (**a**) Electrophoretic mobility shift assays (EMSAs) with DNA-binding mutants of FadR$_{Sa}$. Mutant proteins were prepared by site-directed mutagenesis using an overlap PCR approach followed by heterologous expression and purification with the same procedure as used for the WT protein. EMSAs were performed with radiolabeled DNA probes representing the high-affinity binding region in the promoter region of *Saci_1106*. Experimental procedures are described in the Methods section. Protein concentrations are expressed in nM units. Nucleic acid populations are indicated as follows: F = free DNA, B1 and B2 = specifically bound DNA, NS = non-specifically bound DNA. (**b**) EMSAs with ligand-binding mutants. In each of the lanes, an identical protein concentration was used. Stearoyl-CoA concentrations are mentioned in μM.

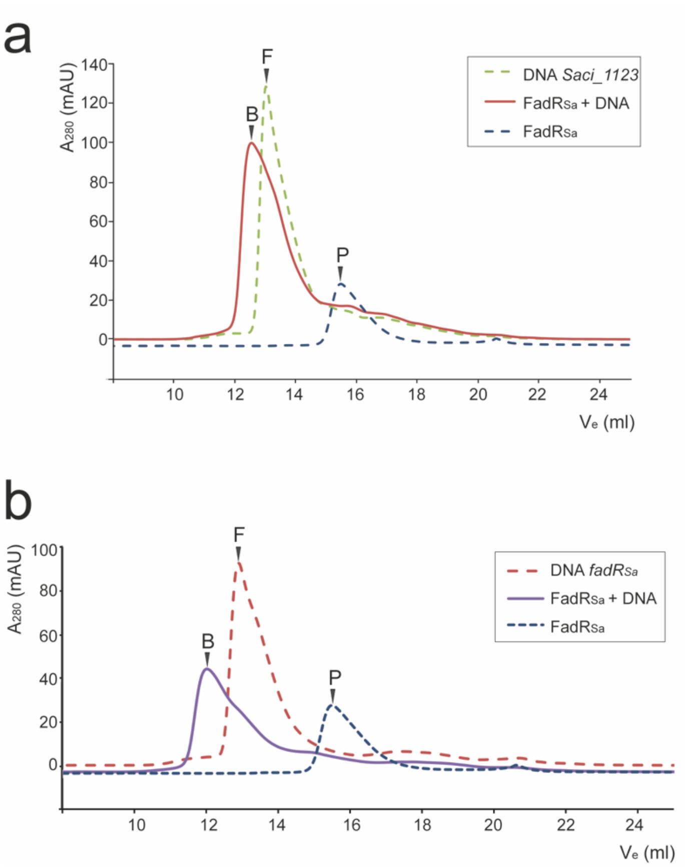

Supplementary Figure 7. Additional SEC analyses of FadR$_{Sa}$-DNA complexes. Size exclusion chromatography (SEC) was performed with 45-bp duplex oligonucleotides harboring the *Saci_1123* (**a**) or *fadR$_{Sa}$* (**b**) operator sequence. Following molar amounts were used: 1 nmol DNA, 8 nmol FadR$_{Sa}$ and 4 nmol FadR$_{Sa}$ mixed with 1 nmol DNA in the case of the *Saci_1123* operator (4:1 molar ratio) or 4 nmol FadR$_{Sa}$ mixed with 0.5 nmol DNA in the case of the *fadR$_{Sa}$* operator (8:1 molar ratio). Peaks are indicated as follows: P = unbound protein, F = unbound DNA and B = protein-DNA complex. Determination of molecular weights of these molecular species is presented in Figure 4a.

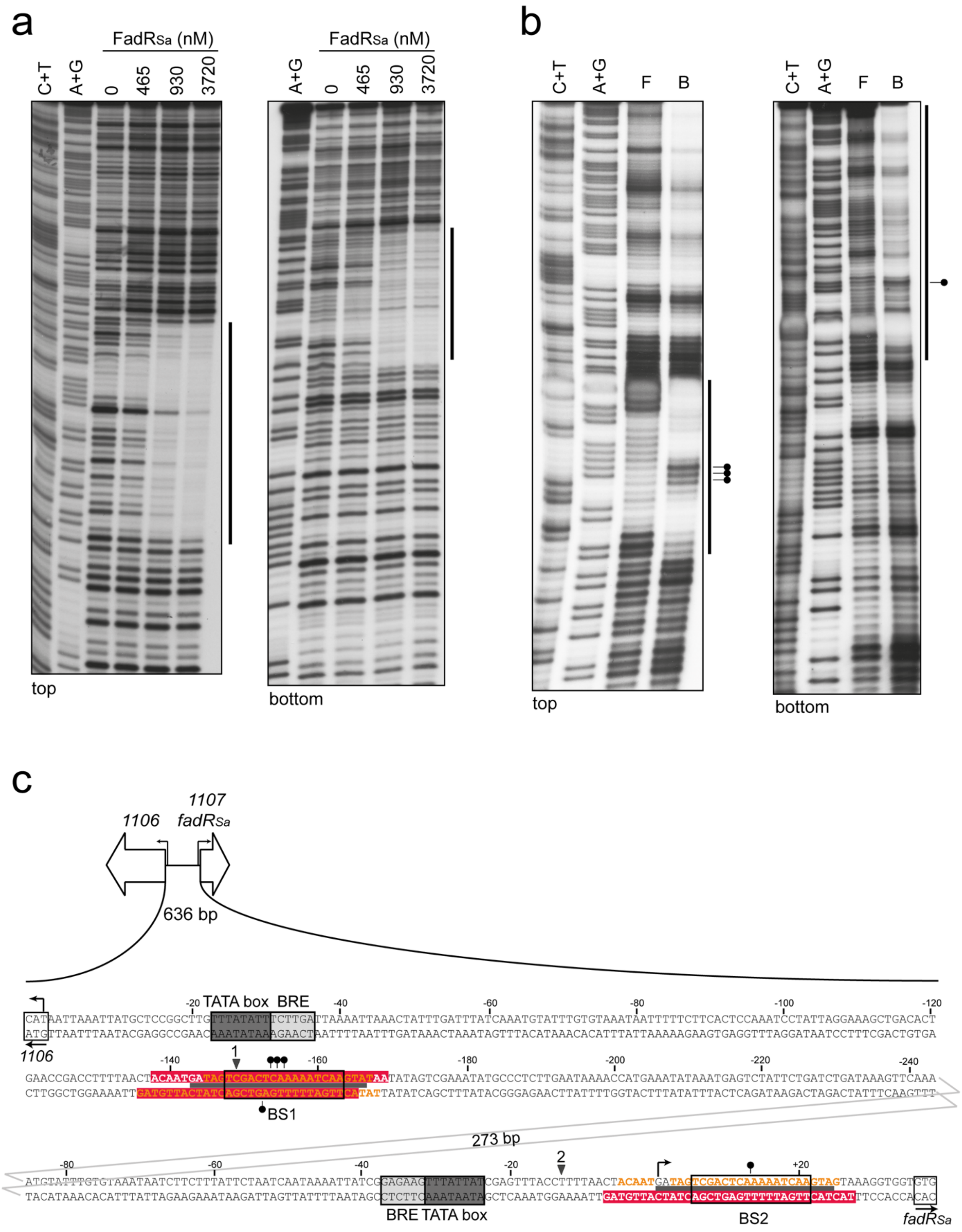

Supplementary Figure 8. *In vitro* contact probing of the interaction between FadR$_{Sa}$ and binding sites in the *Saci_1106-Saci_1107* intergenic region. (**a**) Autoradiographs of DNase I footprinting experiments analyzing FadR$_{Sa}$ binding to a probe representing peak 1 in the *Saci_1106-Saci1107* intergenic region. This was performed for a top-strand and bottom-strand labeled fragment (defined with respect to the orientation of *fadR$_{Sa}$* transcription), as indicated. A+G and C+T denote purine- and pyrimidine-specific Maxam-Gilbert sequencing ladders, respectively. Protected zones are indicated with a vertical line next to the autoradiographs. (**b**) Autoradiographs of chemical ’in-gel’ Cu-phenantroline (Cu-OP) footprinting experiments analyzing FadR$_{Sa}$ binding to a probe representing peak 1. F and B indicate free and bound DNA populations, with the bound population corresponding to the slowest migrating complex in EMSA (B2) (Figure 4b and Supplementary Figure 4b). We do not consider the upper part of the footprint autoradiograph of the experiment with the top strand

labeled as a region protected by protein for two reasons: i) this protection is not observed in the corresponding region when performing the experiment with the bottom strand labelled (see right-hand panel of the figure), while all other footprinting protection zones are confirmed by protection observed for both DNA strands; ii) a nonspecific decrease in band intensity for the larger fragments can be explained by the chemical footprinting reaction conditions leading to excessive DNA cleavage in this specific experiment. Additionally, for the bottom-strand labeled fragment delineation of the protection zone is only accurate for the 5' end. (**c**) Nucleotide sequence of the *Saci_1106-Saci_1107* intergenic region with indication of the protection zones and hyperreactivity sites identified in the footprinting experiments presented in panels (**a**) and (**b**) (peak 1), and in Figure 3e (peak 2). Transcription start sites are indicated with an arrow and based on the observations in [5]. Translational start codons, putative TATA box and factor B recognition element (BRE) promoter elements are boxed, as well as the predicted pseudopalindromic binding sites (BSs). Residues corresponding to the ChIP-seq peak summits are indicated with triangle symbols. White letters in a red background represent protection zones observed in DNase I footprinting experiments, whereas orange letters represent protection zones observed in Cu-OP footprinting experiments. Ball-and-stick symbols represent hyperreactivity effects. A 24-bp repeat that harbors each of the binding sites is indicated with a grey line in between the top and bottom strand.

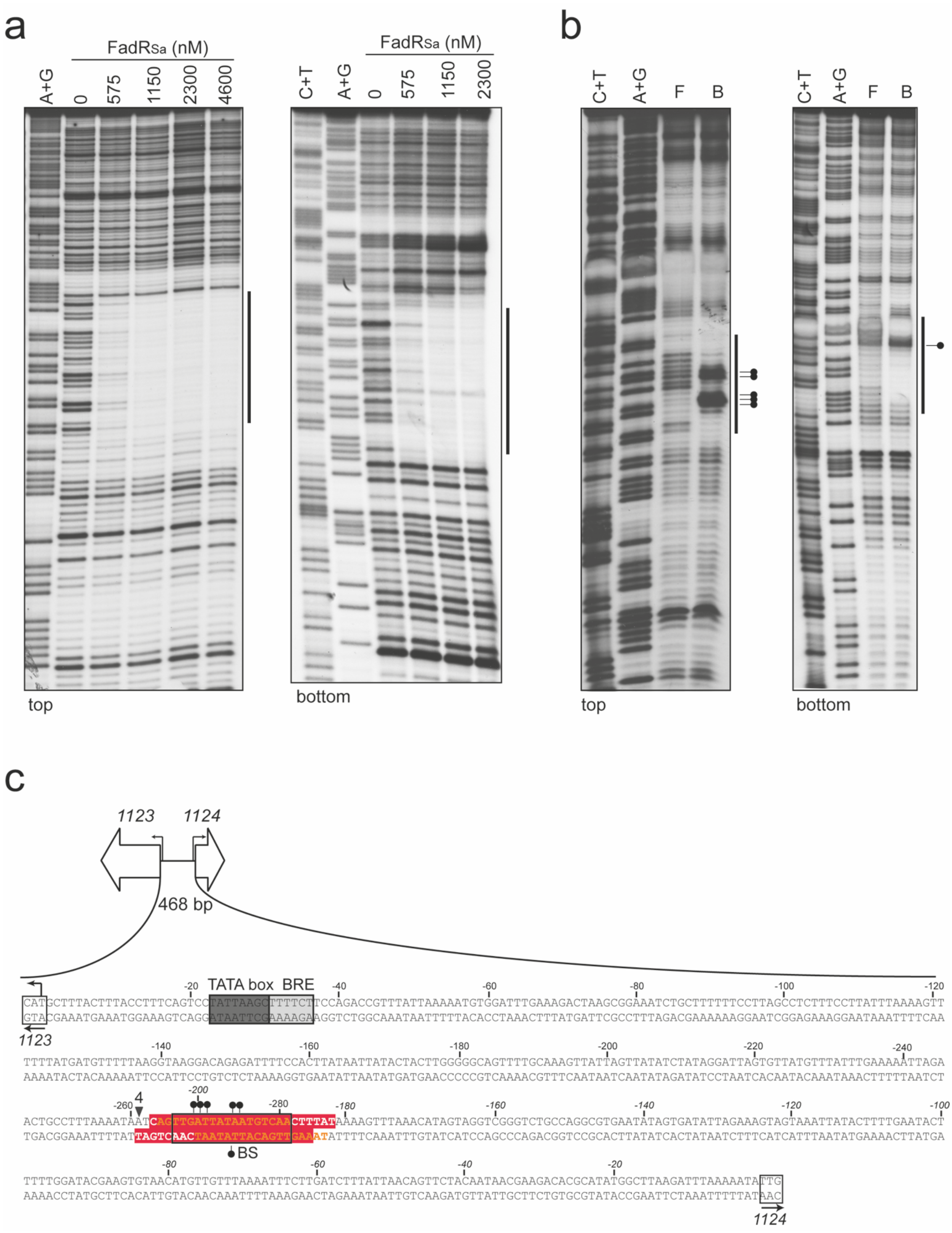

Supplementary Figure 9. ***In vitro*** **contact probing of the interaction between FadR$_{Sa}$ and its binding site in the** ***Saci_1123-Saci_1124*** **intergenic region.** (**a**) Autoradiographs of DNase I footprinting experiments analyzing FadR$_{Sa}$ binding to a probe representing peak 4 in the *Saci_1123-Saci1124* intergenic region. This was performed for a top-strand and bottom-strand labeled fragment (defined with respect to the orientation of *Saci_1124* transcription), as indicated. A+G and C+T denote purine- and pyrimidine-specific Maxam-Gilbert sequencing ladders, respectively. Protected zones are indicated with a vertical line next to the autoradiographs. (**b**)

Autoradiographs of chemical 'in-gel' Cu-phenantroline (Cu-OP) footprinting experiments analyzing $FadR_{Sa}$ binding to a probe representing peak 1. F and B indicate free and bound DNA populations. (**c**) Nucleotide sequence of the *Saci_1123-Saci_1124* intergenic region with indication of the protection zones and hyperreactivity sites as identified in the footprinting experiments presented in panels (**a**) and (**b**). The *Saci_1123* transcription start site is indicated with an arrow and based on the observations in[5]. Translational start codons, putative TATA box and factor B recognition element (BRE) promoter elements are boxed, as well as the predicted pseudopalindromic binding site (BS). Numbering with respect to the *Saci_1123* transcription start site is in regular font while that with respect to the *Saci_1124* start codon is in italic font. The residue corresponding to the ChIP-seq peak summit is indicated with a triangle symbol. White letters in a red background represent protection zones observed in DNase I footprinting experiments, whereas orange letters represent protection zones observed in Cu-OP footprinting experiments. Ball-and-stick symbols represent hyperreactivity effects.

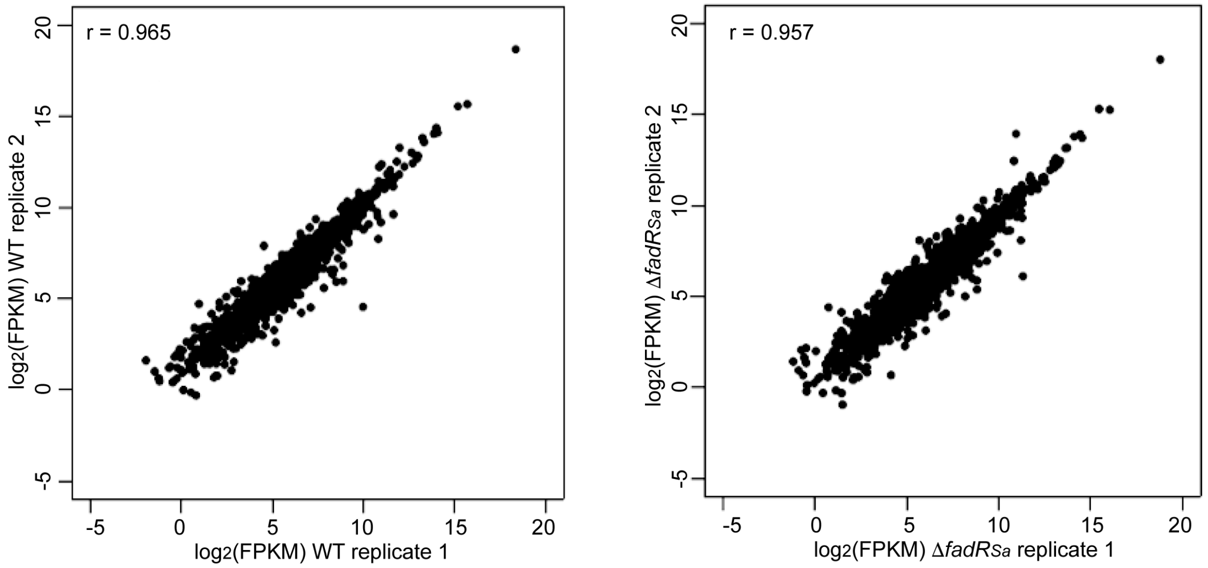

Supplementary Figure 10. Reproducibility of the RNA-seq data. Comparison of the log_2(FPKM) values of the data points between each of the replicates, both for the wild-type samples as for the *ΔfadR$_{Sa}$* samples.

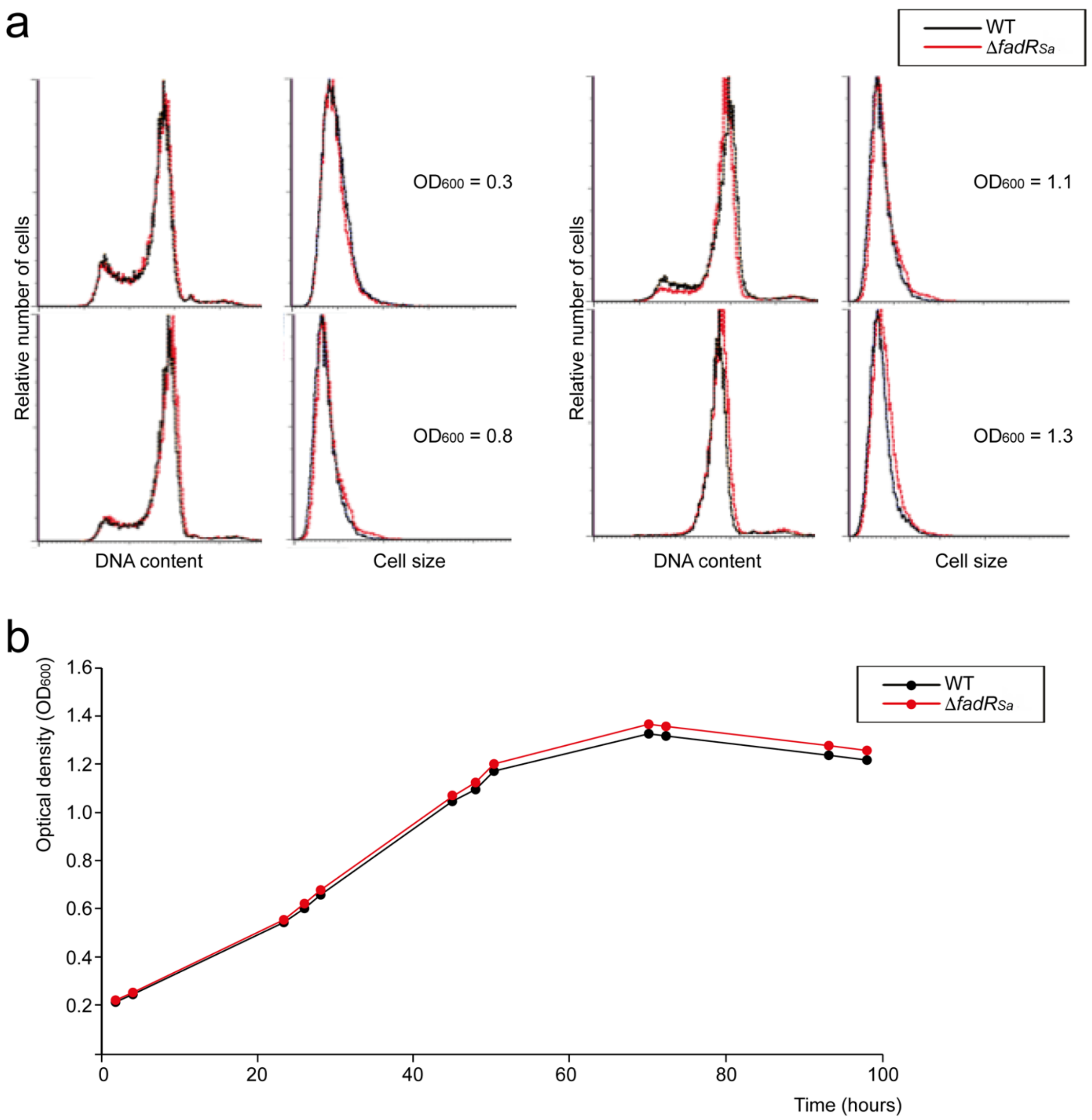

Supplementary Figure 11. Cell morphology and growth behaviour of the FadR$_{Sa}$ deletion mutant. (**a**) Flow cytometry histograms of DNA content and cell size for MW001 and MW001 Δ*fadR$_{Sa}$* strains in different growth phases. Cell samples were collected at four time points during cell culturing representing early (OD_{600} = 0.3), middle (OD_{600} = 0.8) and late (OD_{600} = 1.1) exponential phase and stationary phase (OD_{600} = 1.3). (**b**) Growth curves of MW001 and MW001 Δ*fadR$_{Sa}$* strains in Brock medium containing NZamine and sucrose. Cultures were grown in 50-ml volumes. This experiment was replicated multiple times; a representative curve is shown.

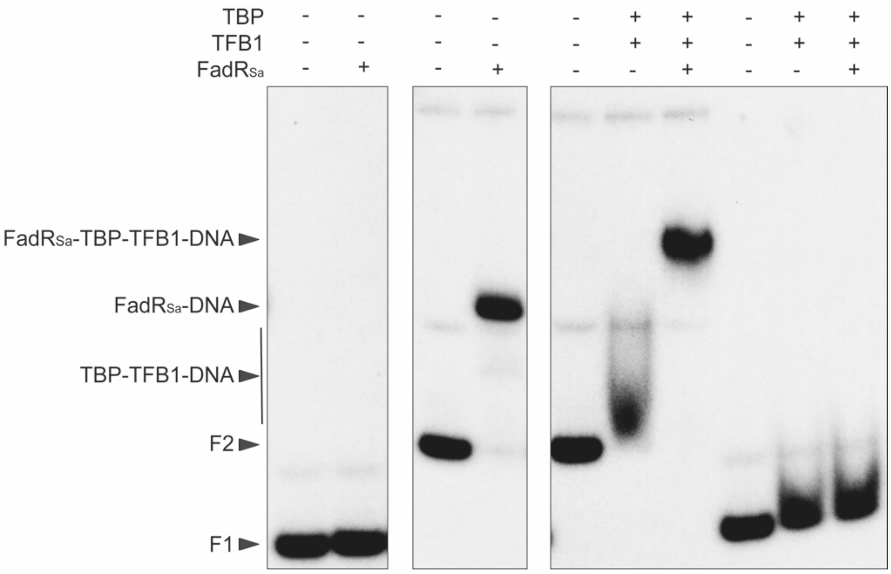

Supplementary Figure 12. *In vitro* DNA-binding analysis of TBP, TFB1 and FadR$_{Sa}$ to the *fadR$_{Sa}$* operator. Electrophoretic mobility shift assay (EMSA) analysis was performed for a radiolabelled negative control probe (F1) encompassing part of an ORF and for a radiolabelled probe containing the *fadR$_{Sa}$* control region (F2). The experimental procedure is described in the Methods section, with supplementation of a 5-fold higher concentration of non-specific competitor DNA (125 ng/μl) to minimize nonspecific interactions between TBP and TFB1 proteins on one hand and DNA on the other hand. Following protein concentrations were used: 974 nM FadR$_{Sa}$, 1.79 μM TBP and 864 nM TFB1. The supplementation of TBP and TFB1 causes the formation of an unstable nucleoprotein complex, while simultaneous addition of TBP, TFB1 and FadR$_{Sa}$ enables the formation of a stable FadR$_{Sa}$-TBP-TFB1-DNA complex with lower migration velocity that that of a FadR$_{Sa}$-DNA complex. This observation points to FadR$_{Sa}$ stimulating TBP and TFB1 binding. As such, the regulator does not employ a repression mechanism in which TBP and TFB1 binding is sterically inhibited, but transcriptional repression can be assumed to occur in later stages of transcription initiation (RNA polymerase binding or open complex formation).

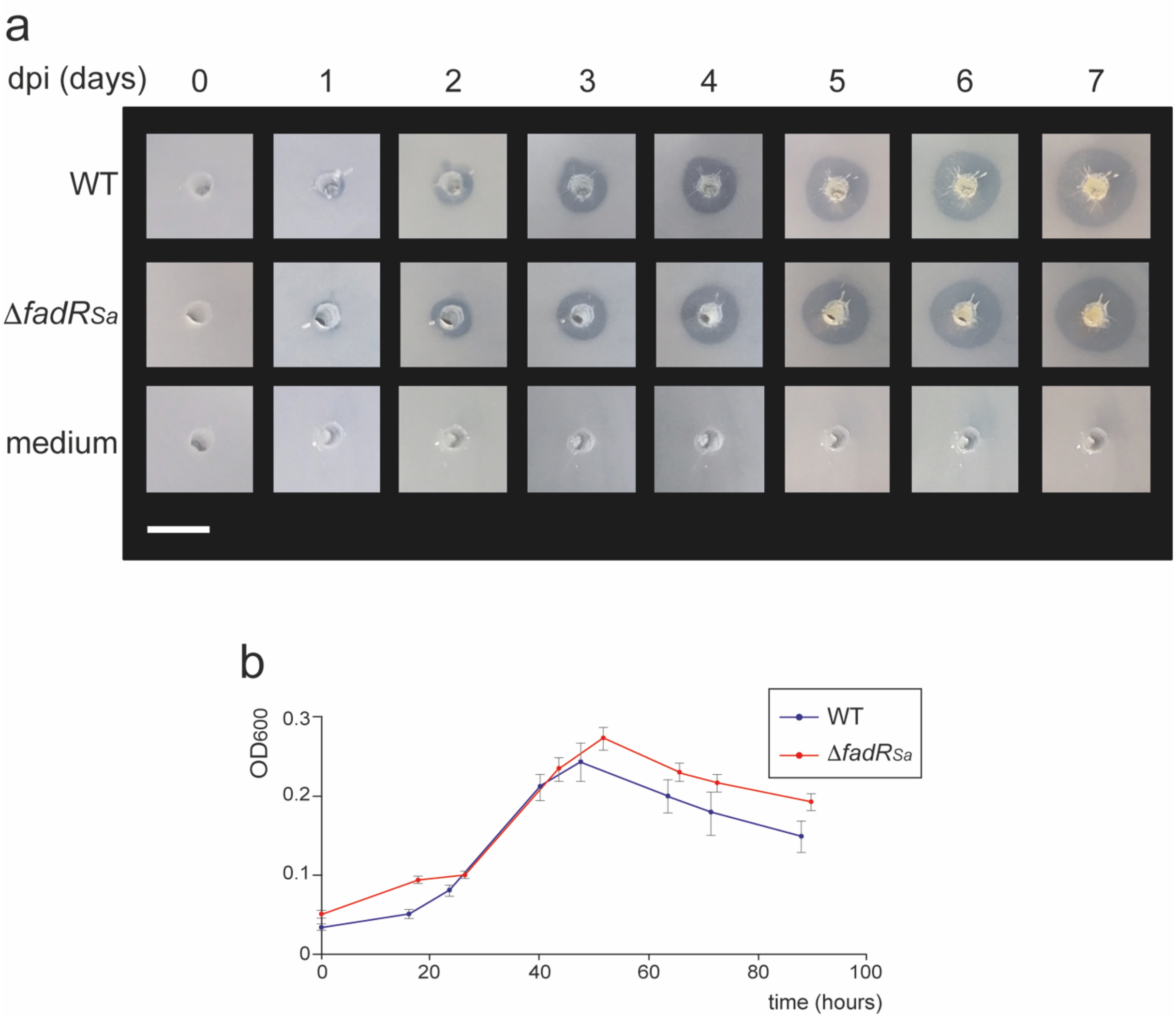

Supplementary Figure 13. Growth behavior of the FadR$_{Sa}$ deletion mutant in the presence of fatty acids and lipids. (**a**) *In vivo* esterase activity assays in which 20 µl of cultures with an OD_{600} around 0.7 were spotted on plates containing 1% (v/v) tributyrin, which were incubated at 78 °C for 7 days. After incubation esterase activity is visible by the appearance of a halo surrounding the original inoculation site indicating the hydrolysis of tributyrin. The esterase plate assay with the *S. acidocaldarius* MW001 (parent strain) and single knockout mutant Δ*fadR$_{Sa}$* strain both show esterase activity. dpi: days post inoculation. The white bar corresponds to 1 cm. (**b**) Growth curves of MW001 and MW001 Δ*fadR$_{Sa}$* strains in Brock medium containing 2 mM butyrate as sole carbon source. Values are averages of four biological replicates with error bars representing standard deviations. Representative curves are shown for multiple independently performed experiments.

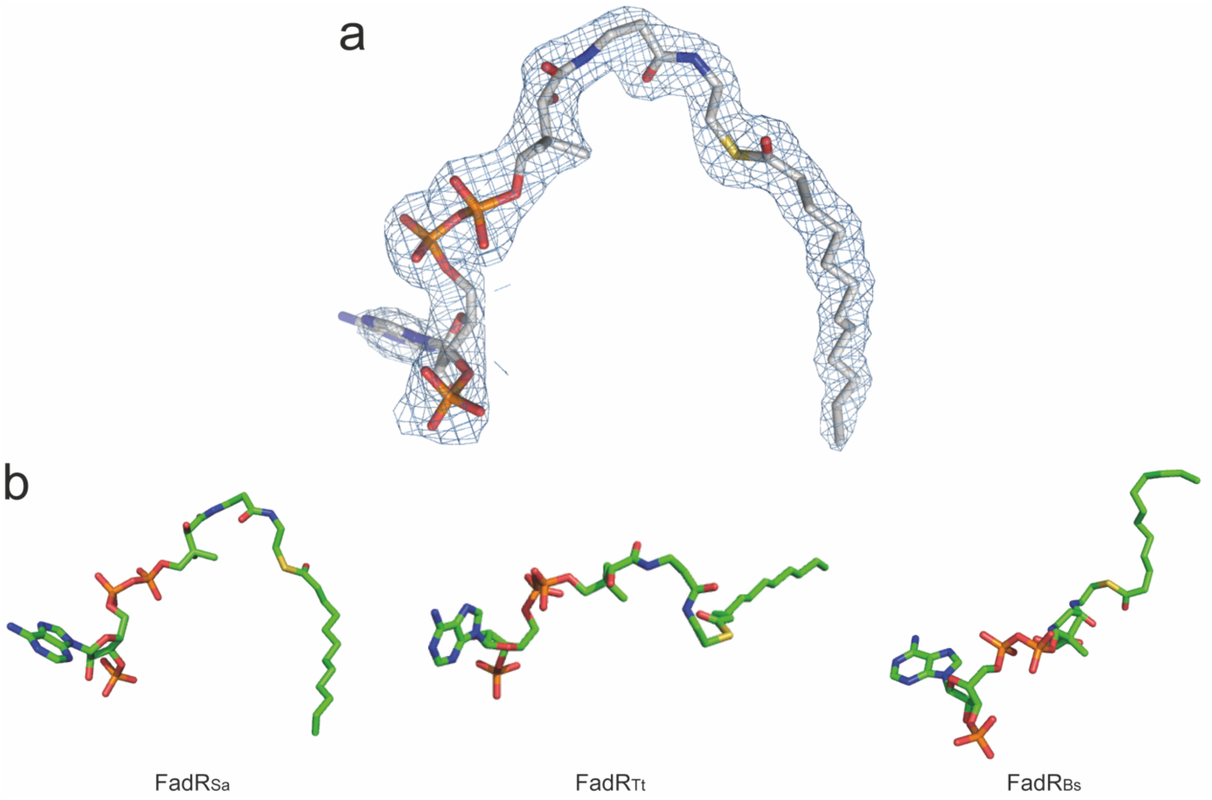

Supplementary Figure 14. Comparison of the ligand conformation in FadR proteins. (**a**) Electron density map of the bound lauroyl-CoA in the $FadR_{Sa}$-lauroyl-CoA cocrystal structure. (**b**) Conformations of lauroyl-CoA in different cocrystal structures of $FadR_{Sa}$ (PDB: 6EL2), $FadR_{Tt}$ (PDB: 3ANG)[6] and $FadR_{Bs}$ (PDB: 3WHB)[7].

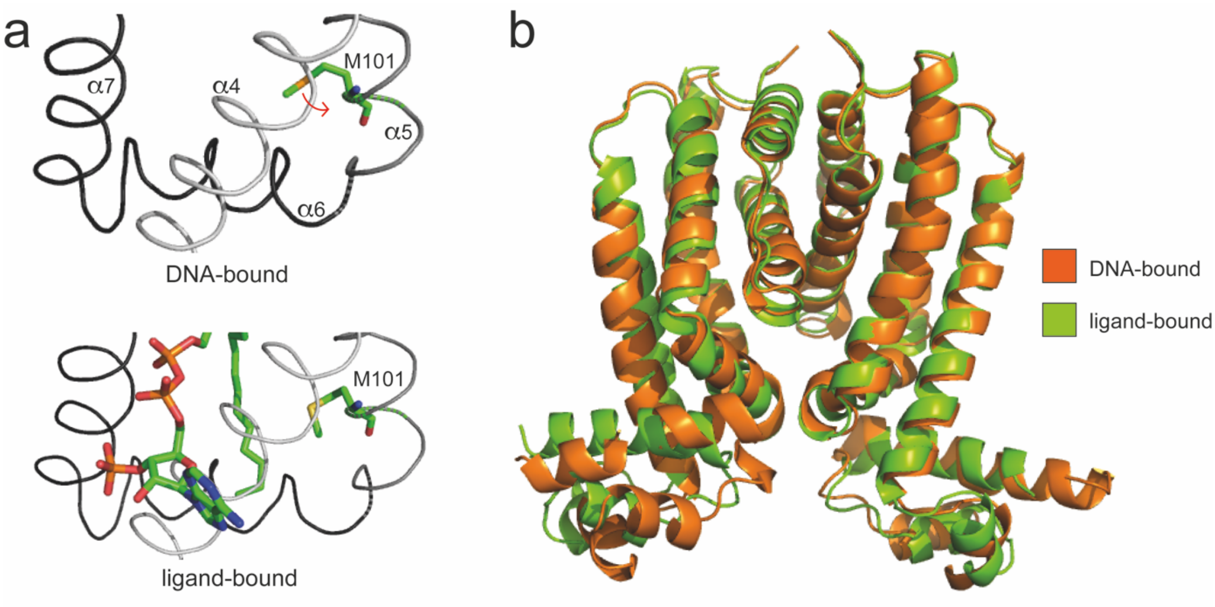

Supplementary Figure 15. Conformational differences between DNA-bound and ligand-bound FadR$_{Sa}$. (**a**) Close-up view of the ligand-binding pocket in DNA-bound and lauroyl-CoA bound FadR$_{Sa}$, with indication of the conformationally altered Met101 residue. (**b**) Superposition of the lauroyl-CoA-bound FadR$_{Sa}$ dimer (green) and subunits A and B of DNA-bound FadR$_{Sa}$ (orange), yielding an RMSD of 1.01 Å considering both subunits in each dimer.

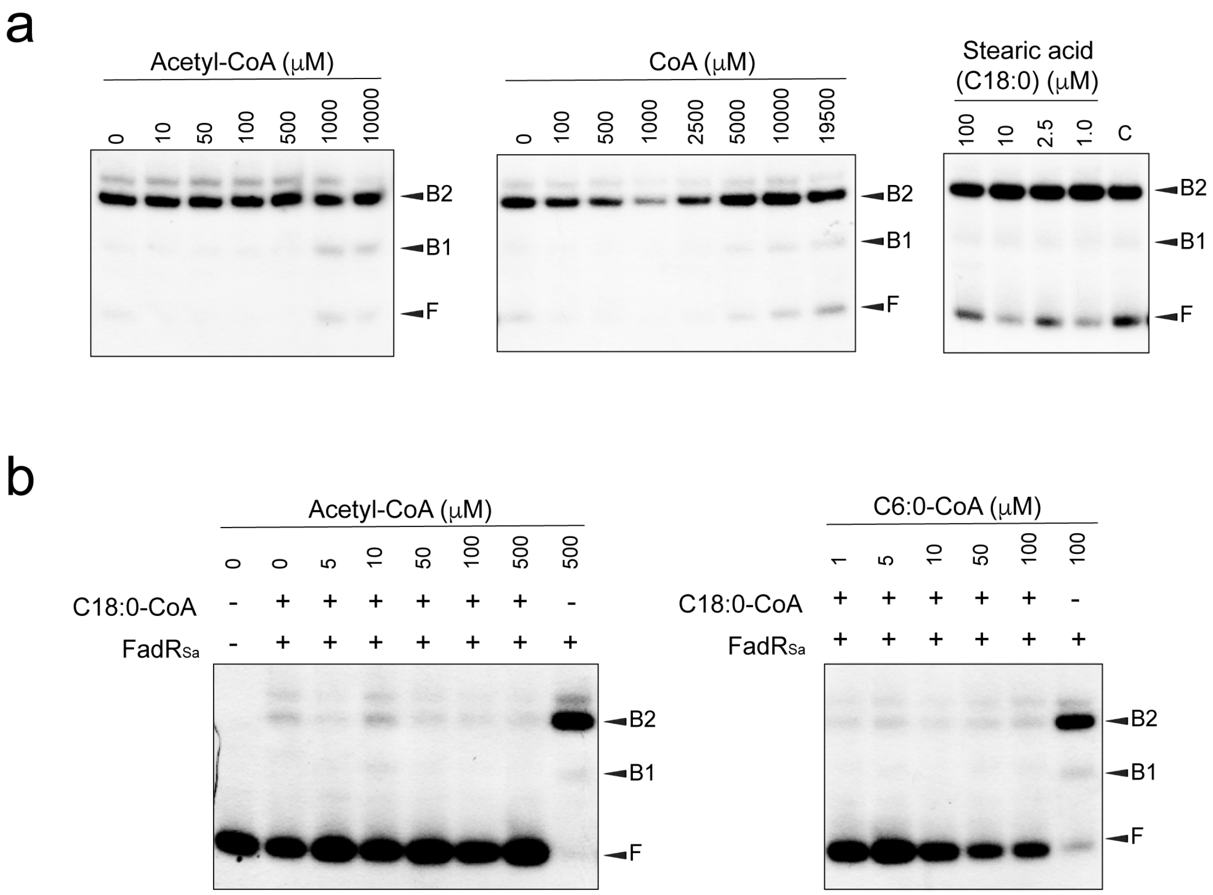

Supplementary Figure 16. Effect of putative ligands on the $FadR_{Sa}$-DNA interaction. Electrophoretic mobility shift assays demonstrating the effect of putative ligands and combinations thereof on the interaction between $FadR_{Sa}$ and a 154-bp radiolabeled DNA probe representing the *Saci_1106* control region. Experimental procedures are described in the Methods section. (**a**) Analysis of the effect of acetyl-CoA, CoA and free fatty acids on the $FadR_{Sa}$-DNA interaction. In all binding reactions, a $FadR_{Sa}$ concentration of 645 nM was applied. Populations of free DNA (F) and of $FadR_{Sa}$-DNA complexes (B1 and B2) are indicated with arrowheads. In some assays, a minor population was observed of an additional nucleoprotein complex migrating somewhat higher than B2. C indicates a control reaction, in which the effect of 2% dimethylsulfoxide (used to dissolve stearic acid) in the binding buffer was tested. Acetyl-CoA and CoA molecules only displayed small ligand-induced inhibition effects at physiologically irrelevant high concentrations of 1 and 5 mM, respectively, while free fatty acids did not affect the $FadR_{Sa}$-DNA interaction at all. (**b**) Competition assay between long- and short-chain acyl-CoA ligands, which were added simultaneously to the binding reactions. In all binding reactions, + indicates the addition of 645 nM $FadR_{Sa}$ or 10 μM stearoyl-CoA, respectively.

Supplementary Table 1. Pairwise amino acid sequence identities and similarities between $FadR_{Sa}$ and bacterial acyl-CoA-dependent TetR members. Following bacterial proteins are considered: $FadR_{Bs}$ of *Bacillus subtilis*, $FadR_{Bh}$ of *Bacillus halodurans*, $FadR_{Tt}$ of *Thermus thermophilus* and $FabR_{Ec}$ of *Escherichia coli*. Alignments were performed with EMBOSS Needle. Values are expressed as %. N-terminal domains are defined as follows: residues 1-55 ($FadR_{Sa}$), 1-48 ($FadR_{Bs}$), 1-49 ($FadR_{Bh}$), 1-52 ($FadR_{Tt}$), 1-72 ($FabR_{Ec}$). C-terminal domains are defined as follows: residues 56-196 ($FadR_{Sa}$), 49-194 ($FadR_{Bs}$), 50-195 ($FadR_{Bh}$), 53-205 ($FadR_{Tt}$), 73-234 ($FabR_{Ec}$).

	**$FadR_{Bs}$**	**$FadR_{Bh}$**	**$FadR_{Tt}$**	**$FabR_{Ec}$**
Identity between FL sequences	26.0	18.8	18.8	17.0
Similarity between FL sequences	41.0	33.2	34.9	33.6
Identity between N-terminal domains	38.2	31.0	27.3	25.7
Similarity between N-terminal domains	58.2	50.0	49.1	43.2
Identity between C-terminal domains	9.0	15.1	11.0	6.6
Similarity between C-terminal domains	16.1	28.1	17.8	15.5

Supplementary Table 2. Summary of ChIP-seq data.

Peak summit coordinate	Fold enrichment	Nearest open reading frame (ORF)	Annotation	Peak summit location[a]	*In vitro* binding[b]	Predicted binding motif	Motif P-value	Distance between motif and summit
191020	2.51	*Saci_0229*	Hypothetical protein	G	++	CTGACTGTAGAATCAA	$6.46E^{-07}$	-1
224943	1.74	*Saci_0266*	Hypothetical protein	G	N.D.	TTGACAAGCTAATCAA	$8.14E^{-06}$	-13
460981	2.21	*Saci_0568*	Hypothetical protein	G	++	TTGAGTCAGTAATCAG	$5.33E^{-06}$	-5
813158	1.74	*Saci_1014*	Aminotransferase	G	N.D.	TTGATACCTGAGTCAA	$6.46E^{-07}$	21
907585	4.24	*Saci_1106*	Acyl-CoA esterase	I	+++	TCGACTCAAAAATCAA	$8.14E^{-06}$	-7
908025	4.24	*Saci_1107*	TetR family transcriptional regulator	I	+++	TCGACTCAAAAATCAA	$8.14E^{-06}$	-26
910364	1.63	*Saci_1109*	Enoyl-CoA hydratase	G	N.D.	N.A.	N.A.	N.A.
918302	2.03	*Saci_1115*	Acyl-2-enoyl-CoA reductase	G	++	TTGACAGAGGGATCAA	$1.19E^{-05}$	-48
928397	2.17	*Saci_1124*	CopG family transcription regulator	I	++	TTGACATTATAATCAA	$2.52E^{-06}$	-14
1161910	1.47	*Saci_1359*	3-hydroxy-3-methylglutaryl coenzyme A reductase	G	N.D.	CTGATAGTATAGTCAA	$7.35E^{-06}$	39
1166706	1.76	*Saci_1364*	Hypothetical protein	I	N.D.	TTGACCCTTTAATCAA	$1.07E^{-06}$	9
1611635	1.83	*Saci_1843*	Cyclase	I	N.D.	TTGATGATATAATCAA	$1.44E^{-06}$	-20
1929449	1.85	*Saci_2107*	Hypothetical protein	G	N.D.	CTGAGTCGGATATCAA	$2.79E^{-05}$	-16
1956504	1.83	*Saci_2130*	Oxidoreductase	G	+	TTGATGCAATGGTCAA	$6.57E^{-06}$	1

[a] G = genic (inside ORF); I = intergenic; [b] *in vitro* binding results.
+ = specific but low-affinity binding, ++ = intermediate-affinity binding, +++ = high-affinity binding.
N.D. = not determined. N.A. = not applicable.

Supplementary Table 3. Binding motif predictions in genomic regions not captured by ChIP-seq.
FIMO predictions of putative $FadR_{Sa}$ binding sites in genomic regions encompassing genes downregulated in the RNA-seq analysis presented in Supplementary Data 2.

**Predicted motif**	**Motif *P*-value**	**Genomic coordinate**	**Location with respect to ORFs**
CTGACCCTTTGGTCAA	$1.71E^{-05}$	2037883	Inside *Saci_2200*
TTGAGAGTGTCCTCAA	$3.19E^{-05}$	1634798	Inside *Saci_1859*
CTGATAGTCAAATCAG	$4.69E^{-05}$	2093127	Inside *Saci_2250*
TCGACAGGAACGTCAA	$8.62E^{-05}$	380550	Inside *Saci_0447*
CTGAAGACATAATCAG	$9.20E^{-05}$	2094465	Inside *Saci_2252*
CTGACTATAGAGTCTA	$9.30E^{-05}$	2039429	Inside *Saci_2201*

FIMO predictions of putative $FadR_{Sa}$ binding sites in the *Saci_1103-Saci_1126* genomic region (genomic coordinates: 902793-932298) not retrieved in previous characterizations and/or predictions presented in Supplementary Table 2.

**Predicted motif**	**Motif *P*-value**	**Genomic coordinate**	**Location with respect to ORFs**
TTGATTCTAGGTTCAA	$3.55E^{-06}$	906727	Inside *Saci_1106*
TCGACCATGGAGTCAA	$8.89E^{-06}$	930823	Inside *Saci_1126*
TCGAGAGGATAATCAG	$6.93E^{-05}$	909604	Inside *Saci_1108*

Supplementary Table 4. Summary statistics and alignment information of the paired-end RNA-seq experiment. WT = *S. acidocaldarius* MW001; KO = *S. acidocaldarius* MW001 Δ*fadR*$_{Sa}$.

Sample	Raw read length (bp)	Read pairs	Mapped read pairs	Mapping rate (%)
WT1	124	8639675	8313946	96.23
WT2	124	10597695	10196655	96.20
KO1	124	11779481	11330497	96.20
KO2	124	7618684	7343047	96.40

Supplementary Table 5. Overview of strains used in this work.

Name	Description/purpose	Reference or source
Escherichia coli DH5α	Plasmid propagation strain	Gibco
E. coli Rosetta (DE3)	Protein overexpression strain	Novagen
E. coli Rosetta 2 (DE3)	Protein overexpression strain	Novagen
E. coli ER1821	Strain used for plasmid methylation	New England Biolabs
Sulfolobus acidocaldarius DSM639	Wild-type strain	DSMZ
S. acidocaldarius MW001	Uracil auxotrophic strain for genetic experiments	[8]
S. acidocaldarius MW001Δ*fadR*	Markerless *fadR* gene deletion mutant	This work

Supplementary Table 6. Overview of plasmids used in this work.

Name of plasmid	Description/purpose	Reference
pET45b	Protein overexpression vector	Novagen
pET24a	Protein overexpression vector	Novagen
pET30a	Protein overexpression vector	Novagen
pET45bx*fadR*$_{Sa}$	pET45b containing the *fadR*$_{Sa}$ open reading frame	This work
pET24ax*fadR*$_{Sa}$*Ndenull*	pET24a containing *fadR*$_{Sa}$*Ndenull*	This work
pET24ax*fadR*$_{Sa}^{Y47A}$*Ndenull*	pET24a containing *fadR*$_{Sa}$*Ndenull* with Y47A mutation	This work
pET24ax*fadR*$_{Sa}^{G48A}$*Ndenull*	pET24a containing *fadR*$_{Sa}$*Ndenull* with G48A mutation	This work
pET24ax*fadR*$_{Sa}^{Y51A}$*Ndenull*	pET24a containing *fadR*$_{Sa}$*Ndenull* with Y51A mutation	This work
pET24ax*fadR*$_{Sa}^{Y53A}$*Ndenull*	pET24a containing *fadR*$_{Sa}$*Ndenull* with Y53A mutation	This work
pET24ax*fadR*$_{Sa}^{R73A}$*Ndenull*	pET24a containing *fadR*$_{Sa}$*Ndenull* with R73A mutation	This work
pET24ax*fadR*$_{Sa}^{R86A}$*Ndenull*	pET24a containing *fadR*$_{Sa}$*Ndenull* with R86A mutation	This work
pET24ax*fadR*$_{Sa}^{M101A}$*Ndenull*	pET24a containing *fadR*$_{Sa}$*Ndenull* with M101A mutation	This work
pET30ax*tbp*	pET30a containing the *tbp* open reading frame	This work
pET30ax*tfb1*	pET30a containing the *tfb1* open reading frame	This work
pSVA431	Backbone plasmid for gene disruption construct, harbouring a *pyrEF* selection marker	[8]
pSVA431x*ΔfadR*$_{Sa}$	Suicide *fadR*$_{Sa}$ gene disruption construct	This work

SUPPLEMENTARY REFERENCES

1. Yeo, H. K., Park, Y. W. & Lee, J. Y. Structural basis of operator sites recognition and effector binding in the TetR family transcription regulator FadR. *Nucleic Acids Res.* **45**, 4244-4254 (2017).
2. Schumacher M. A. *et al.* Structural basis for cooperative DNA binding by two dimers of the multidrug-binding protein QacR. *EMBO J.* **21**, 1210-1218 (2002).
3. Itou H., Watanabe, N., Yao, M., Sirakihara, Y. & Tanaka, I. Crystal structures of the multidrug binding repressor *Corynebacterium glutamicum* CgmR in complex with inducers and with an operator. *Journal of Molecular Biology* **403**, 174-184 (2010).
4. Yang S. *et al.* Structural basis for interaction between *Mycobacterium smegmatis* Ms6564, a TetR family master regulator, and its target DNA. *J. Biol. Chem.* **288**, 23687-23695 (2013).
5. Cohen, O. *et al.* Comparative transcriptomics across the prokaryotic tree of life. *Nucleic Acids Res.* **44,** W46–53 (2016).
6. Agari, Y., Agari, K., Sakamoto, K., Kuramitsu, S. & Shinkai, A. TetR-family transcriptional repressor *Thermus thermophilus* FadR controls fatty acid degradation. *Microbiology* **157,** 1589–1601 (2011).
7. Fujihashi, M. *et al.* Structural characterization of a ligand-bound form of *Bacillus subtilis* FadR involved in the regulation of fatty acid degradation. *Proteins* **82,** 1301–1310 (2014).
8. Wagner, M. *et al.* Versatile genetic tool box for the crenarchaeote *Sulfolobus acidocaldarius*. *Front Microbiol* **3,** 214 (2012).
