# Supplementary material for: A TetR-family transcription factor regulates fatty acid metabolism in the archaeal model organism Sulfolobus acidocaldarius
Source: Nat Commun. 2019 Apr 4;10:1542. doi: 10.1038/s41467-019-09479-1 (PMC6449355; doi:10.1038/s41467-019-09479-1)
Supplement: Supplementary file 3 — Description of Additional Supplementary Files [file 41467_2019_9479_MOESM3_ESM.pdf]

Description of Additional Supplementary Files

File Name: Supplementary Data 1
Description: Overview of FadRSa-DNA interactions identified in the cocrystal structure for one of the DNA duplexes (XY)

File Name: Supplementary Data 2
Description: Summary of RNA-seq data

File Name: Supplementary Data 3
Description: Overview of the FadRSa-lauroyl-CoA interactions identified in the ligandbound cocrystal structure of the subunits (subunit B)

File Name: Supplementary Data 4
Description: Overview of oligonucleotides used in this work
