# Supplementary material for: A TetR-family transcription factor regulates fatty acid metabolism in the archaeal model organism Sulfolobus acidocaldarius
Source: Nat Commun. 2019 Apr 4;10:1542. doi: 10.1038/s41467-019-09479-1 (PMC6449355; doi:10.1038/s41467-019-09479-1)
Supplement: Supplementary file 8 — Reporting Summary [file 41467_2019_9479_MOESM8_ESM.pdf]

nature research

Corresponding author(s): Ann-Christin Lindas, Eveline Peeters

Last updated by author(s): Feb 19, 2019

Reporting Summary

Nature Research wishes to improve the reproducibility of the work that we publish. This form provides structure for consistency and transparency in reporting. For further information on Nature Research policies, see Authors & Referees and the Editorial Policy Checklist.

Statistics

For all statistical analyses, confirm that the following items are present in the figure legend, table legend, main text, or Methods section.

n/a	Confirmed	
☐	☒	The exact sample size (n) for each experimental group/condition, given as a discrete number and unit of measurement
☐	☒	A statement on whether measurements were taken from distinct samples or whether the same sample was measured repeatedly
☒	☐	The statistical test(s) used AND whether they are one- or two-sided<br>*Only common tests should be described solely by name; describe more complex techniques in the Methods section.*
☒	☐	A description of all covariates tested
☒	☐	A description of any assumptions or corrections, such as tests of normality and adjustment for multiple comparisons
☒	☐	A full description of the statistical parameters including central tendency (e.g. means) or other basic estimates (e.g. regression coefficient) AND variation (e.g. standard deviation) or associated estimates of uncertainty (e.g. confidence intervals)
☒	☐	For null hypothesis testing, the test statistic (e.g. F, t, r) with confidence intervals, effect sizes, degrees of freedom and P value noted<br>*Give P values as exact values whenever suitable.*
☒	☐	For Bayesian analysis, information on the choice of priors and Markov chain Monte Carlo settings
☒	☐	For hierarchical and complex designs, identification of the appropriate level for tests and full reporting of outcomes
☒	☐	Estimates of effect sizes (e.g. Cohen's d, Pearson's r), indicating how they were calculated

Our web collection on statistics for biologists contains articles on many of the points above.

Software and code

Policy information about availability of computer code

Data collection	No software was used.
Data analysis	The following software was used for data analysis (either commercial or open-source): for structure determination and refinement: XDS program package, PHENIX, O, Phaser, PDBsum and PyMOL; for ChIP-seq analysis: Burrows-Wheeler Aligner BWA 0.7.10, MACS2 (2.1.0), IGV version 2.3.59, BEDTools and MEMEsuite; for RNA-seq analysis: cutadapt 1.9.1, Tophat 2.0.12, Cufflinks 2.2.1, Subread package 1.5.0 and DESeq2; for qRT-PCR analysis: Prism 6.0 (Graphpad).

For manuscripts utilizing custom algorithms or software that are central to the research but not yet described in published literature, software must be made available to editors/reviewers. We strongly encourage code deposition in a community repository (e.g. GitHub). See the Nature Research guidelines for submitting code & software for further information.

Data

Policy information about availability of data

All manuscripts must include a data availability statement. This statement should provide the following information, where applicable:

- Accession codes, unique identifiers, or web links for publicly available datasets
- A list of figures that have associated raw data
- A description of any restrictions on data availability

All crystal structures presented in this work have been deposited in the Protein Data Bank (PDB) and are available with accession codes 5MWR [http://dx.doi.org/10.2210/pdb5MWR/pdb, has been superseded with 6EL2] (native FadRSa structure), 6EL2 [http://dx.doi.org/10.2210/pdb6EL2/pdb] (lauroyl-CoA-bound FadRSa structure) and 6EN8 [http://dx.doi.org/10.2210/pdb6EN8/pdb] (DNA-bound FadRSa structure). All raw data for the ChIP-seq and RNA-seq studies presented in this work have been deposited in the Gene Expression Omnibus (GEO) databank with accession codes GSE108039 [https://www.ncbi.nlm.nih.gov/bioproject/?term=GSE108039] and GSE108018 [https://www.ncbi.nlm.nih.gov/bioproject/?term=GSE108018], respectively.

Field-specific reporting

Please select the one below that is the best fit for your research. If you are not sure, read the appropriate sections before making your selection.

☒ Life sciences ☐ Behavioural & social sciences ☐ Ecological, evolutionary & environmental sciences

For a reference copy of the document with all sections, see nature.com/documents/nr-reporting-summary-flat.pdf

Life sciences study design

All studies must disclose on these points even when the disclosure is negative.

Sample size	No sample-size calculation was performed, sample sizes were chosen based on similar molecular microbiology studies previously published.
Data exclusions	No data were excluded from the analyses.
Replication	Experimental designs included biological and/or technical replicates as described in the Methods section: the ChIP-seq and ChIP-qPCR experiments were performed for biological duplicates, EMSAs were performed in technical replicates, qRT-PCR was performed in biological triplicates and technical duplicates.
Randomization	This is not relevant to our study, since it involves a single microbial strain.
Blinding	This is not relevant to our study, since it involves a single microbial strain.

Reporting for specific materials, systems and methods

We require information from authors about some types of materials, experimental systems and methods used in many studies. Here, indicate whether each material, system or method listed is relevant to your study. If you are not sure if a list item applies to your research, read the appropriate section before selecting a response.

Materials & experimental systems

n/a	Involved in the study	
☐	☒	Antibodies
☒	☐	Eukaryotic cell lines
☒	☐	Palaeontology
☒	☐	Animals and other organisms
☒	☐	Human research participants
☒	☐	Clinical data

Methods

n/a	Involved in the study	
☐	☒	ChIP-seq
☒	☐	Flow cytometry
☒	☐	MRI-based neuroimaging

Antibodies

Antibodies used	Customized antibodies were used: FadRSa-specific antibodies (produced by immunizing a rabbit with purified recombinant FadRSa (Innovagen)).
Validation	Validation of the antibodies was performed by ChIP-qPCR (presented in Figure 2b in the main manuscript).

ChIP-seq

Data deposition

☒ Confirm that both raw and final processed data have been deposited in a public database such as GEO.

☐ Confirm that you have deposited or provided access to graph files (e.g. BED files) for the called peaks.

Data access links<br>*May remain private before publication.*	https://www.ncbi.nlm.nih.gov/bioproject/?term=GSE108039
Files in database submission	Saci_1107_ChIPSeq_rep1, Saci_1107_ChIPSeq_rep2, input DNA_rep1, input DNA_rep2, Mock_IP_rep1, Mock_IP_rep2
Genome browser session<br>(e.g. UCSC)	no longer applicable

Methodology

Replicates	ChIP-seq experiments were performed in biological duplicates with input and mock controls.
Sequencing depth	Sequencing was performed in single-end with read length of 51bp. Duplicate IP samples have 1580665 reads (1304493 uniquely mapped) and 3606763 reads (3376176 uniquely mapped), respectively. Duplicate Input samples have 1155563 reads (1136744 uniquely mapped) and 1826999 reads (1602096 uniquely mapped), respectively. Duplicate Mock samples have 712496 reads (654952 uniquely mapped) and 121700 reads (106319 uniquely mapped), respectively.
Antibodies	Customized antibodies were used: FadRSa-specific antibodies (produced by immunizing a rabbit with purified recombinant FadRSa (Innovagen)). Rabbit serum that used for mock experiments was collected before immunization (Innovagen).
Peak calling parameters	Command line: macs2 callpeak -t ChIP.bam -c control.bam -f BAM -B -g 2300000 --nomodel --extsize 200
Data quality	Peaks are called with with 0.05 qvalue cut-off(FDR), and no peaks have a fold-enrichment higher than 5.
Software	Bcl to Fastq conversion was performed using bcl2Fastq v1.8.3 from the CASAVA software suite; sequence reads were mapped to the Sulfolobus acidocaldarius DSM639 genome (NC_007181.1) with Burrows-Wheeler Aligner (BWA 0.7.10) with default parameters; peaks were called using MACS2 (2.1.0); IGV version 2.3.59 was used to browsing the ChIP-seq results; BEDTools and MEMEsuite were used to determine the binding motifs.
